# Examining the effectiveness of home-based cardiac rehabilitation programs for heart failure patients with reduced ejection fraction: a critical review

**DOI:** 10.1186/s12872-023-03640-x

**Published:** 2023-12-05

**Authors:** Shahram Darvishzadehdaledari, Alexander Harrison, Fatemeh Gholami, Arian Azadnia

**Affiliations:** 1 Department of Health Sciences, University of York, York, UK; 2 Department of Health Sciences, University of York, York, UK; 3 Department of Epidemiology, School of Public Health, Iran University of Medical Sciences, Tehran, Iran; 4 Social Determinants of Health Research Center, Research Institute for Health Development, Kurdistan University of Medical Sciences, Sanandaj, Iran; 5 Research and Technology Deputy, Maragheh University of Medical Sciences, Maragheh, Iran

**Keywords:** Heart failure, Cardiac rehabilitation, Home-based, Center-based, ESC, BACPR

## Abstract

**Background:**

Heart failure (HF) is the most common cardiovascular reason for hospital admission, particularly among patients older than 60 years old. Heart failure with reduced ejection fraction (HFrEF) comprises approximately 50% of all heart failure cases. Home-based cardiac rehabilitation (HBCR) is an alternative option to enhance the participation rate in cardiac rehabilitation (CR) interventions for patients who are not able to attend center-based cardiac rehabilitation (CBCR). The purpose of this review is to clarify the extent to which present studies of HBCR align with the core components defined by both the European Society of Cardiology (ESC) and the British Association for Cardiac Prevention and Rehabilitation (BACPR).

**Methods:**

A critical review was conducted through four databases, MEDLINE, Embase, Cochrane Central Register of Controlled Trials, and Cochrane Database of Systematic Reviews, to identify randomized controlled trials up until June 2022. We scrutinized the commonalities between BACPR and ESC and developed a list of standards. The risk of bias was assessed using the RoB 2 tool.

**Results:**

Among the 87 papers selected for full-text screening, 11 studies met the inclusion criteria. Six papers possessed a high proportion of fidelity to essential standards, four studies had a medium alliance, and one intervention had a low level of alliance.

**Conclusion:**

Overall, the majority of included studies had medium to high alignment with standards and core components. However, a need for more attention to long-term strategy as an important standard is revealed. Rapid identification and initial assessment are the most met standards; however, lifestyle risk factor management and long-term outcomes were recognized as the least met standards.

**Supplementary Information:**

The online version contains supplementary material available at 10.1186/s12872-023-03640-x.

## Introduction

Heart failure (HF) stands out as the most common cardiovascular reason for hospital admission, especially among patients aged 60 and older [1]. Patients with HF are typically classified into three main groups: 1) reduced ejection fraction (LVEF < 40%), 2) mid-range ejection fraction (LVEF 40–49%), and 3) preserved ejection fraction (LVEF ≥50%) [2]. HF remains a growing global epidemic, affecting over 37.7 million individuals worldwide [3, 4]. Heart failure with reduced ejection fraction (HFrEF) constitutes approximately 50% of all heart failure cases in the United States and is associated with significant morbidity, diminishing the quality of life [5]. HFrEF places a substantial healthcare burden, particularly for patients requiring rehospitalization or urgent outpatient care for heart failure, despite adhering to medical therapy based on established guidelines [6].

Centre-based cardiac rehabilitation (CBCR) is a supervised group-based intervention conducted in sports centers or hospitals [7, 8]. Cardiac rehabilitation (CR) is a recognized multidisciplinary intervention involving exercise, education, encouragement of physical activity, heart-related risk management, and mental support, tailored to patients diagnosed with cardiovascular diseases [9]. Extensive research confirms the safety and effectiveness of medically supervised CBCR programs, playing a crucial role in reducing hospital readmission, mortality, and subsequent cardiac events, historically until COVID and 2020 [10–14]. However, despite improvements in referral rates to CR centers, participation rates remain low among various demographic groups, attributed to factors such as travel time, lack of availability due to work commitments, reluctance to engage in group therapies, and the impact of COVID-19 [15–18]. Survey findings on CR barriers reveal that both women and men face numerous obstacles, including high costs, perceiving exercise as tiring or painful, long distances, and facility-related responsibilities [19].

Home-based cardiac rehabilitation (HBCR) is recognized as a practical approach to address barriers faced by CBCRs, implemented in nonclinical settings such as homes, clubs, community centers, and parks [20]. A systematic review and meta-analysis suggested that HBCR interventions using wearable sensors can be as effective as CBCR, significantly boosting participation rates, adherence, and accessibility in CR interventions by overcoming various participation obstacles [21]. Recent research, including a 2022 review, indicates that HBCR, as an alternative to CBCR, is considered safe and feasible [22]. According to European guidelines on CVD prevention, the emergence of HBCR has significantly increased participation rates in preventative programs [23].

The European Society of Cardiology (ESC) and the British Association for Cardiovascular Prevention and Rehabilitation (BACPR) provide crucial guidelines to prevent the increasing burden of cardiovascular-related mortality [24, 25]. Both guidelines emphasize that CR programs should encompass core components and standards for maximum effectiveness, including physical activity counseling, patient assessment, diet/nutritional counseling, patient education, exercise training, risk factor control, psychosocial management, and long-term outcome audit and evaluation [26, 27].

Currently, there is a growing body of evidence from trials assessing the effectiveness of HBCR for HF patients. However, the extent to which interventions in these studies align with national and international guidance varies. Therefore, the aim of this study is to review the evidence critically and assess the degree to which each study aligns with these standards, with the key objective being to determine ‘how’ this alignment is achieved.

## Method

Critical review in the context of the quality of research and fidelity of study interventions against clinical standards and core components for CVD prevention and rehabilitation based on ESC and BACPR. First, we systematically searched and screened the literature, reviewed BACPR and ESC to develop a comprehensive list of the core elements of CR, and then critically assessed each included study against the new list for core elements.

We intended to implement this review according to the PRISMA (Preferred Reporting Items for Systematic Reviews and Meta-Analysis) statement and Cochrane Handbook for interventions [28, 29].

### Eligibility criteria

Eligibility criteria were described in the context of the population and intervention. Heart failure with reduced ejection fraction (HFrEF) is the population type for the study. HBCR, known as a structured program, was the obvious intervention. Interventions focusing on only one specific muscle, those based on physiotherapy or not mentioning CR, were excluded. In terms of study type, randomized control trials (RCTs) (cluster or individual level) will be included.

### Search strategy

MEDLINE, EMBASE, Cochrane CDSR, and the Cochrane Central Register of Controlled Trials (CENTRAL) databases were assessed until July 01, 2022. MeSH terms in the MEDLINE and other synonyms have been used as keywords (see more details in Additional file 1, pages 1–14). Language and data limits were not applied. The structure of the searches was as follows: Heart Failure OR myocardial failure OR Heart decompensation OR myocardial decompensation AND Cardiac Rehabilitation OR heart rehabilitation OR home-based rehabilitation AND hospital-based OR standard care OR usual care AND effectiveness OR cost-effectiveness AND randomized controlled trail OR randomized trial OR controlled trials. The PRISMA flow diagram was used for the inclusion and exclusion criteria assessment [28]. Two reviewers (R1) and (R2) assessed the inclusion and exclusion criteria for selecting studies. A third reviewer (P.D.) provided an independent view of the papers to resolve discrepancies. We checked the reference lists of all included studies and relevant systematic reviews to identify additional studies missed during the original electronic searches. Furthermore, we contacted the authors of identified studies for information on unpublished or ongoing trials or to request additional data.

### Data extraction

Numerous information categories, such as study design, publication year, location, information about the intervention (intervention type, duration, frequency and intensity), baseline characteristics (e.g., age, sex), duration of follow-up, and characteristics of the control group, were obtained within the extraction process. All papers recruited patients based on the NYHA classification [30].

### Essential components of CR

Since there are many items explaining the same content among both groups of standards (ESC and BACPR), we decided to scrutinize the commonality among them (Table 1). Researchers selected determined components (AH and SD). Delivering the intervention by a well-experienced team is introduced as the first component, followed by rapid identification, recognition of eligible patients, and provision of a well-structured prevention program as the second component. Then, patients’ needs based on their preferences should be assessed, and appropriate care must be available at this stage. Managing physical activity, body composition, healthy diet, smoking cessation, and other lifestyle risk factors are considered the next component. Psychological support, medical risk management, and exercise training are predominantly common between BACPR and ESC. Assessing long-term outcomes is considered another key component. Based primarily on the BACPR core components, inclusion and engagement with national audits is considered a core standard. This is because it can play an important role in both improving the quality of delivered services and informing national policy [31]. In 2016, in the UK, the NCP_CR was launched as a result of coordination between BACPR and NACR aiming at ensuring that all CR programs meet the minimum standards [32]. The audit and evaluation aspect of the BACPR core components has been long standing; however, trials do not need to mention audits as they collect/analyze and report on their own collected data; moreover, audits are a local service aspect and may be different between countries. Accordingly, we did not consider audit as a common component in this study. After scrutinizing the commonalities among both ESC and BACPR, we reached a comprehensive list of essential standards (Table 2).

**Table 1:** Essential components of cardiac rehabilitation

BACPR		ESC
Standards	Core-components	Core-components
1) Identification and referral	1) Health behavior change and education.	1) Patient assessment with medical control
2) Multidisciplinary team	2) Lifestyle risk factor management: physical activity and exercise training - healthy eating and body composition - tobacco cessation and relapse prevention	2) Physical activity counseling
3) Initial assessment	3) Psychosocial health	3) Exercise training
4) Delivery of programme	4)Medical risk management	4) Diet/nutritional counseling
5) Final assessment	5) Long-term strategies	5) Risk factor control
6) Audit and evaluation		6) Patient education
		7) psychosocial management
		8) vocational advice

**Table 2:** Essential components of cardiac rehabilitation considering both BACPR & ESC

1. Delivery of the intervention by a well-experienced and multidisciplinary team	
2. Rapid identification, early provision of a structured cardiovascular prevention and rehabilitation programme, and recruitment of eligible individuals	
3. Initial assessing of patient’s needs and provide cares that meets patient’s preferences and purposes	
4. Lifestyle risk factor management - physical activity, healthy eating, body composition, tobacco cessation and relapse prevention	
5. Psychological health and management	
6. Medical risk management	
7. Comprehensive exercise	
8. Long-term strategy and illustration of a constant health outcome	

### Assessing the essential components

We evaluated the alignment of interventions with standards and components. Concerning the delivery of CR interventions by a multidisciplinary team, if three or more medical professionals were involved, the CR program fulfilled this component. Conversely, papers with two or fewer medical staff members were deemed ineligible for meeting this criterion. Lifestyle risk factor management was categorized into physical activity, healthy eating (nutritional advice), and tobacco cessation. CR programs monitoring two or three lifestyle risk management practices met this component, while studies monitoring only one were considered unmet. Assessing BP, glucose, lipids, HR, implantable cardiac devices (ICD), and current medication use were deemed essential for controlling medical risk factors. Papers assessing three or more risk factors were considered to implement a standard intervention; however, studies investigating two or fewer risk factors did not meet the standard. Interventions assessing medication usage, as it enables easy monitoring of crucial risk factors, were classified as meeting the risk factor management component. Papers providing screening plans or treatment procedures for psychological issues met the psychological support standard. The initial assessment component needed the evaluation of comorbidities, psychological status, QoL, physical status, baseline characteristics, hemodynamic parameters, and medical history at baseline. For a long-term strategy, papers adhered to BACPR and ESC if they had a follow-up period of at least 1 year. Interventions prescribing individualized exercise training aligned with the comprehensive exercise element and the inclusion of resistance exercise alongside aerobic exercise were recommended. Studies were categorized based on their level of alliance: low (three or fewer components), medium (four to five), or high (six or above). using the author’s determined cutoff that corresponds to current reporting from the National Audit of Cardiac Rehabilitation [33], BACPR [34], and participation in the National Certification Programme (NCP_CR) [32]. Notably, there are no peer-reviewed published articles covering this aspect.

### Risk of Bias 2 (ROB2) assessment

We employed the updated Cochrane tool (2019) to assess the risk of bias or measure the quality of clinical trial methodology [29]. Compared to its 2011 edition, the new version is more complex, requiring researchers to possess basic knowledge of clinical trial study design, conduct, and analysis standards. The tool applies to all three types of randomized trials: parallel groups, cluster randomized trials, random crossover, and other matched designs [29].

The new tool comprises five domains, each containing several questions with five response options. Judgment bias of risk was assigned at the end of each domain, categorized as low risk of bias, some concerns, or high risk of bias according to Cochrane guidelines. A final section, “overall bias,” sums up all five domains, leading to the study being classified as one of the three categories mentioned above. Bias domains are detailed in additional file 1, page 15.

## Results

### Description of included papers

**Fig. 1:**
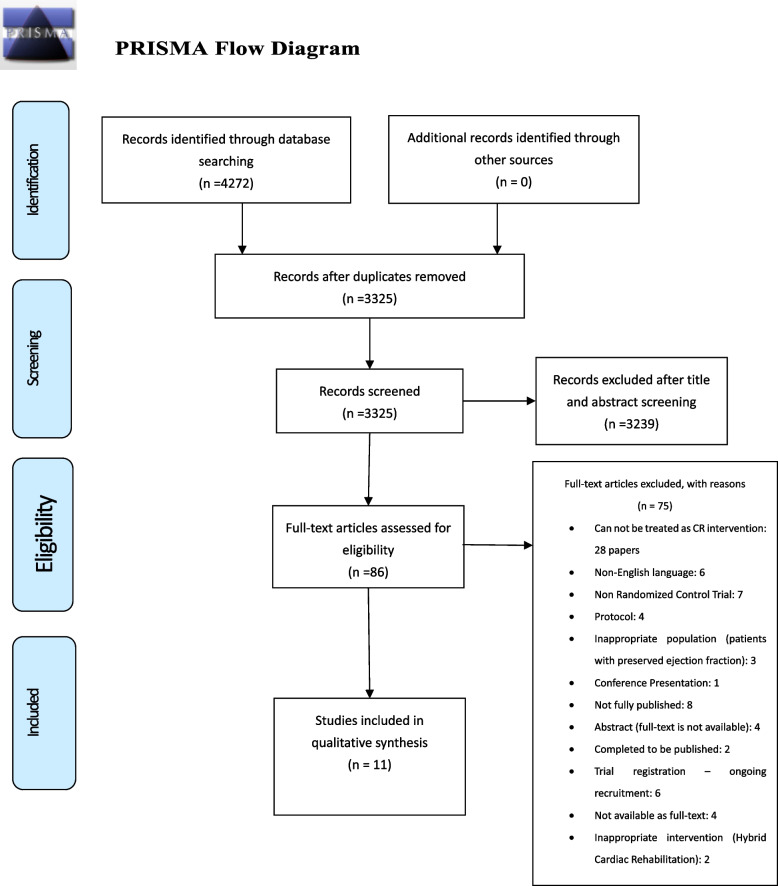
Flow diagram of the study selection process

 A total of 4272 papers were identified through database searches. After deduplication, 3325 unique records remained. Among the selected papers, 3239 studies were excluded during abstract and title screening due to lack of relevance. Subsequently, 86 studies were identified as potentially suitable for full-text screening, leading to the final inclusion of 11 papers (Fig. 1). Of these, three studies were conducted in Poland, two in the UK, two in China, and one each in Turkey, Japan, Taiwan, and Canada. The systematic review included 1241 participants, with study sizes ranging from 30 to 216 participants. The participants had a mean age ranging from 31.58 to 80.6. The control groups varied, with hospital-based cardiac rehab and CBCR serving as controls in two papers, while other control groups primarily received usual care. The majority of papers reported outcomes over 3 months, with two studies having a 12-month follow-up. Program durations ranged from 8 weeks to 12 months, with variations in session lengths (20 to 60 minutes) and frequencies (one to five times per week) (Table 3).

**Table 3:** Characteristics of studies included on cardiac rehabilitation

*Study*	*Study Design*	*Number of prticipants*	*Age group*	*Intervention*	*Follow-up*	*City/* *Country*	*NYHA*
***Piotrowicz*** **et al.*2010*** [35]	Prospective randomized trial	152	Aged 58.1 + 10.2 years	Home-based vs SCR	8 weeks after randomization	Warsaw, Poland	Yes Class II or III
***Karapolat*** **et al.** ***2009*** [36]	Randomized control trial	74	Home-based: 45.16 ± 13.58 Hospital-based: 44.05 ± 11.49	Home-based vs hospital-based	8 weeks after randomization	Izmir, Turkey	Yes Class II or III
***Ma*** **et al.*2022*** [37]	A prospective randomized controlled trial	136	Intervention group: 64.18 ± 8.70 Control group: 63.56 ± 8.29	Home-based vs community Care	24 weeks after randomization	Guangzhou, southern China	Yes Class I, II and III
***Nagatomi*** **et al.** ***2022*** [38]	Single-center, open-label, randomized, controlled trial	30	63.7 ± 10.1 years	Home-based vs standard care	3 months after randomization	Fukuoka, Japan	Yes Class I, II and III
***Chen*** **et al.** ***2018*** [39]	Randomized prospective trial	37	Control group: 60 ± 16 Intervention group: 61 ± 11	Home-based vs standard medical care	3 months after randomization	Taichung, Taiwan	Yes Not specified
***Frost*** **et al.** ***2019*** [40]	Multicenter randomized controlled trial	216	Intervention group: 69.7 ± 10.9 Control group: 68.5 ± 9.8	REACH-HF + usual Care vs usual Care	12 months after randomization	Four geographical regions (Birmingham, Cornwall, Gwent and York) across the UK	Yes Class II or III
***Peng*** **et al.** ***2018*** [41]	Prospective, randomized control trial	98	Years≤60 = 30.6% Years> 60 = 69.4%	Home-based telehealth exercise training program vs usual care	4 months after randomization	Chengdu, Republic of China	Yes Class I, II and III
***Piotrowicz*** **et al.** ***2015*** [42]	Single-center, prospective, parallel-group, randomized (2:1), controlled trial	111	Intervention group: 54.4 ± 10.9 Control group: 62.1 ± 12.5	Home-based telemonitored NW vs usual Care	8 weeks after randomization	Warsaw, Poland	Yes Class II and III
***Dalal*** **et al.** ***2019*** [43]	Multicenter, two parallel group, randomized, superiority trial	216	Intervention group: 69.7 ± 10.9 Control group: 69.9 ± 11	REACH-HF vs usual care	12 months after randomization	Four centers in the United Kingdom (Birmingham, Cornwall, Gwent and York)	Yes Class I, II, III and IV
***Safiyari-Hafizi*** **et al.** ***2016*** [44]	Randomized control trial	40	< 75 years	HBCR vs usual care	12 weeks after randomization	British Columbia, Canada	Yes Not specified
***Piotrowicz*** **et al.** ***2019*** [45]	Prospective randomized controlled trial	131	56.4 ± 10.9	Home-based telemonitored cardiac rehabilitation vs standard cardiac rehabilitation	8 weeks after randomization	Warsaw, Poland	Yes Class II and III

### Risk of bias assessment

**Fig. 2:**
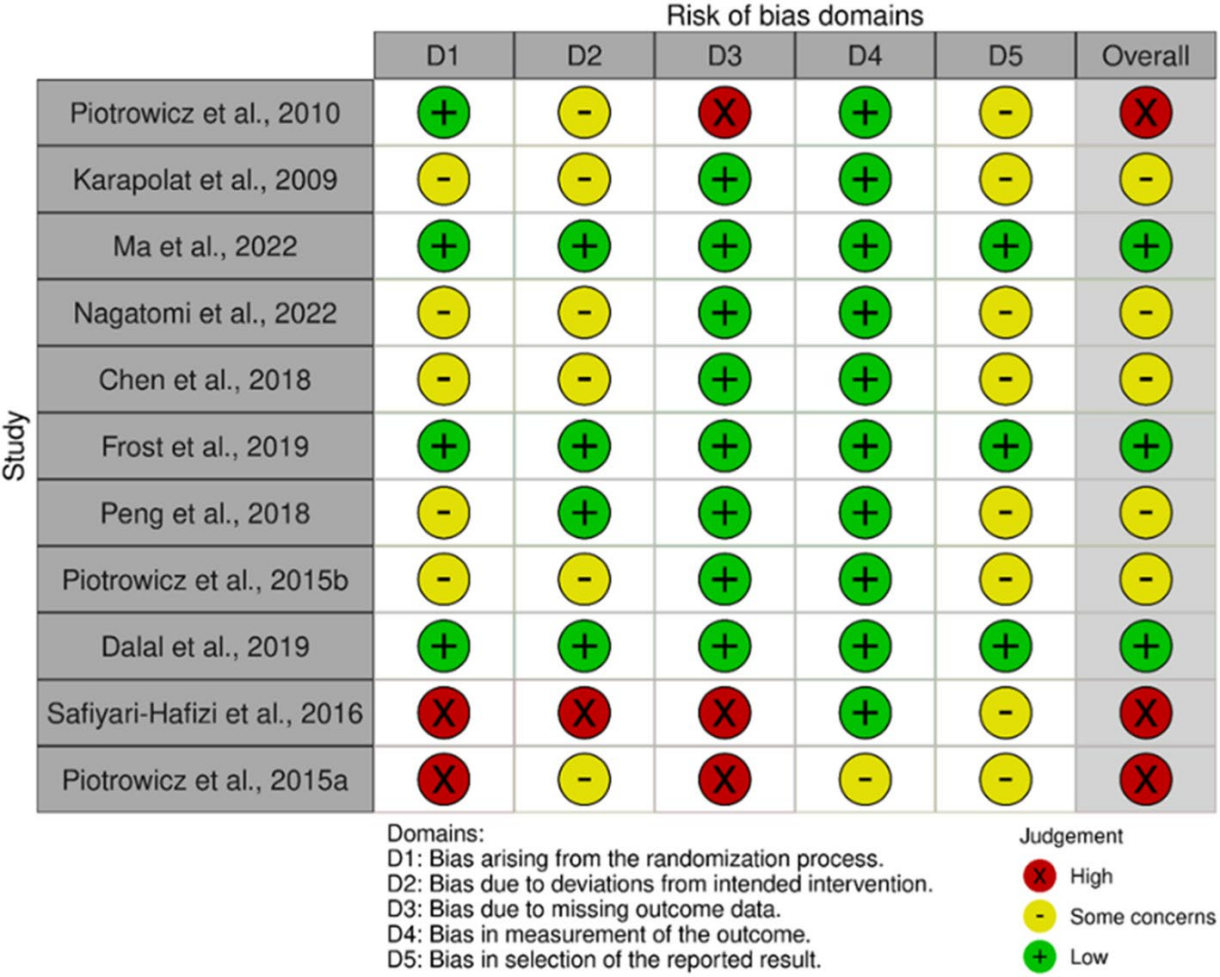
Risk of bias 2 assessment

Eleven studies underwent quality evaluation. Among them, three studies (27.275%) were classified as having a low risk of bias, five studies (45.45%) fell into the some concerns group, and three studies (27.275%) were categorized as having a high risk of bias (refer to Fig. 2). Notably, there was insufficient detail to accurately assess methodological quality, hindering the ability to determine bias. Evaluating selection bias proved challenging due to limited information on random allocation sequence generation and concealment in most reports. While many studies exhibited deviations from the intended intervention, these deviations were generally balanced between groups. However, there is a potential impact on the study outcome due to this deviation.

### Essential components of HBCR interventions

**Fig. 3:**
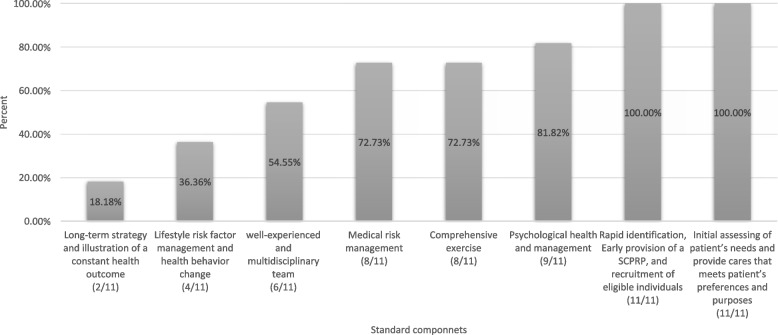
Percentage of alliance with essential components of cardiac rehabilitation considering both BACPR & ESC

In Fig. 3, rapid identification and initial assessment of patient needs are recognized as standards in all included studies (100%). Psychological management follows with 81.82%, while medical risk management, rapid identification, and comprehensive exercise share the same percentage (72.73%). Well-experienced teams and lifestyle risk factor management accounted for 54.55 and 36.36%, respectively. The long-term strategy is identified as the least met standard (18.18%). Table 4 presents essential components of HBCR interventions, with each component explained separately.

**Table 4:** Proportion of alliance with Essential components of cardiac rehabilitation considering both BACPR & ESCs

Study	Well-experienced and multidisciplinary team	Rapid identification, early provision of a SCPRP, and recruitment of eligible individuals	Initial assessing of patient’s needs and provide cares that meets patient’s preferences and purposes	Lifestyle risk factor management and health behavior change	Psychological health and management	Medical risk management	Comprehensive exercise	Long-term strategy and illustration of a constant health outcome	Total
**Piotrowicz et al.2010 **[35]	Yes	Yes	Yes	No	Yes	Yes	Yes	No	6
***Karapolat*** **et al.** ***2009*** [36]	No	Yes	Yes	No	Yes	Yes	Yes	No	5
***Ma*** **et al.*2022*** [37]	Yes	Yes	Yes	Yes	Yes	Yes	Yes	No	7
***Nagatomi*** **et al.** ***2022*** [38]	Yes	Yes	Yes	Yes	No	Yes	Yes	No	6
***Chen*** **et al.** ***2018*** [39]	Yes	Yes	Yes	No	No	No	Yes	No	4
***Frost*** **et al.** ***2019*** [40]	No	Yes	Yes	Yes	Yes	Yes	No	Yes	6
***Peng*** **et al.** ***2018*** [41]	Yes	Yes	Yes	No	Yes	No	Yes	No	5
***Piotrowicz*** **et al.** ***2015*** [42]	No	Yes	Yes	No	Yes	Yes	Yes	No	5
***Dalal*** **et al.** ***2019*** [43]	Yes	Yes	Yes	Yes	Yes	Yes	No	Yes	6
***Safiyari-Hafizi*** **et al.** ***2016*** [44]	No	Yes	Yes	No	Yes	No	No	No	3
***Piotrowicz*** **et al.** ***2019*** [45]	Yes	Yes	Yes	No	Yes	Yes	Yes	No	6

### Delivery of the intervention by a well-experienced and multidisciplinary team

In the majority of papers, the type and number of staff involved in the intervention were clearly described. Employing a multidisciplinary team, two studies [35, 46] included five different professionals in each study: a cardiologist, nurses, general practitioners, certified trainers, physiotherapists, ECG technicians, and psychologists. In terms of nutritional management and guidance, four studies were noted [36–39]. Nagatomi et al. stood out for recruiting numerous dietitians [36]. Peng et al. [40] utilized three medical staff, including a physiotherapist, cardiac nurse, and psychiatrist, while other studies benefited from using two different professionals [41–43]. Nurses, physiotherapists, and cardiologists were predominantly recruited in most studies. Only two papers exclusively used psychologists and other mental health professionals to screen and treat psychological disorders [40, 44]. Piotrowicz et al. [39] is the only paper with no specified multidisciplinary team.

### Rapid identification, early provision of a structured cardiovascular prevention and rehabilitation programme, and recruitment of eligible individuals

Eligible participants should be recruited during inpatient stays and after discharge. Encouraging all eligible patients to participate in CR programs during hospitalization is essential [31]. Patients with HFrEF were the eligible population in all papers reviewed. Most studies recruited admitted patients in hospitals or medical centers, with one study inviting a combination of hospitalized and discharged patients [37]; four different geographical regions were involved in recruitment in two papers [38, 42]. In one study, leaflets were distributed in community health centers, inviting interested HF patients to contact the team to participate [35].

### Initial assessment of patient needs and providing care that meets patient preferences and purposes

All 11 included papers used an initial assessment for study participants. Physical examination tests included the 6-minute walking test (6MWT), cardiopulmonary exercise treadmill test (CPET), pulmonary function test, physical performance testing (PPT), 30-s arm curl test, 10 sit-stand-to-sit test, and short physical performance battery (SPPB) performance score. QOL was measured using the Minnesota Living with Heart Failure Questionnaire (MLHFQ). Comorbidities, cardiovascular events, previous surgeries, stroke, and smoking history were considered when assessing past medical history. Functional status was assessed using the NYHA classification and measuring LVEF. Approximately half of the papers specified the medication taken. Mental health status was assessed using the Beck Depression Inventory, Hospital Anxiety and Depression Scale (HADS), and Spielberger State-Trait Anxiety Inventory [41, 42].

### Lifestyle risk factor management - physical activity, healthy eating, body composition, tobacco cessation, and relapse prevention

Performing and maintaining regular exercise were emphasized by the exercise support team in one study [35], with recommendations provided to prevent patients from engaging in dangerous exercises such as swimming in another separate study [42]. Information about eating a balanced diet and managing weight was not accurately discussed in the included papers for this study. Nevertheless, one study recruited several dieticians responsible for providing useful advice on nutritional guidance [36]. Moreover, numerous strategies to monitor weight were presented in [38]. While all selected papers did not refer to smoking behavior changes as a reported outcome, two studies assessed the history of any tobacco use [38, 41]. Additionally, one paper provided smoking cessation for patients’ partners [42].

### Psychological health and management

Most included studies for this review (9 out of 11) took necessary measurements to provide either comprehensive screening (diagnosis) for detecting any mental issues or strategies and approaches aiming to improve or treat current psychological issues. Two papers did not introduce any psychological programs during the intervention [36, 37], and five papers provided psychological support for patients [35, 39, 42, 44, 46]. Levels of anxiety and depression were assessed using tools including the HADS, Beck Depression Inventory, and Spielberger State-Trait Anxiety Inventory [38, 40–42].

### Medical risk management

Although there was no medication management (changing the doses and medication titration) during the interventions, current medication was assessed among most of the included studies. The presence of ICDs was specified in two studies [38, 46], and participants with an ICD were considered differently in one paper [46]. During CR interventions, HR was regularly monitored in the majority of papers. ECG was monitored in three studies [39, 44, 46]. and controlling BP was assessed in three papers [36, 44, 46]. The level of total cholesterol was measured during HBCR in one particular study [36].

### Comprehensive exercise

All 11 papers included in this study involved exercise training. Heart rate reserve (HRR), ranging from 40 to 80%, and Borg score range (BSR), ranging from 11 to 14, were the main assessors to determine the proportion of exercise intensity. Treadmills, cycle ergometers, walking, and jogging were the main models of exercise implemented by the majority of studies. Among most interventions, exercise training frequency ranged from three times per day to five sessions per week, and each session lasted between 20 and 60 minutes. Among the selected papers for this review, four articles did not include warming-up and cooling-down sections (3 to 10 min) [36–38, 43]. Only a simple individualized walking program was utilized as an exercise program in one study [43]; furthermore, two types of exercises, a chair-based exercise and progressive walking without frequency, time, intensity, and duration of exercise or with these parameters (frequency, duration, and intensity) arranged by patients, were delivered as a training program [38, 42]. Although the third of these papers by Thomas et al. had an exercise program during the implementation of the CR, they cannot be considered a comprehensive exercise program due to reasons such as lack of individualized intensity, duration, and frequency of exercise based on patients’ ability and lack of warm-up and cool-down sections. The majority of HBCR interventions involved walking with a wide range of support through home visits or phone calls arranged by mainly physical therapists and nurses. It is obvious that the majority of participants will be given the most appropriate physical activity (walking or jogging) in accordance with their capacity and ability; however, several obstacles, including comorbidities or logistical concerns (lack of access to a gym or an inappropriate surface), may impose a negative effect on the ultimate effectiveness of HBCR; accordingly, new approaches or strategies are needed to increase the beneficial effects of HBCR [20].

### Long-term strategy and illustration of a constant health outcome

The majority of chosen papers (9 out of 11) in this study have a follow-up time ranging between 8 weeks and 6 months; thus, patients cannot be followed up, leading to a limitation in detecting long-term effects. However, there are two studies assessing the long-lasting (12 months) impacts of CR intervention on patients [38, 42].

## Discussion

Various studies have indicated that HBCR and CBCR exhibit comparable effectiveness and adherence. However, HBCR programs not only demonstrate similar effectiveness but also showcase superior cost-effectiveness [45, 47]. This review seeks to assess the alignment of HBCR programs for HFrEF patients with the standards and core components outlined by both ESC and BACPR. The results reveal variable levels of adherence, ranging from low to high across the included studies. Six papers exhibit a high fidelity to essential standards [35, 36, 38, 39, 42, 46], while four studies demonstrate a medium level of adherence [37, 40, 41, 44]; only one intervention displays a low level of fidelity [43]. The rapid identification and recruitment of eligible individuals, as well as the initial assessment of patients, are two essential standards fully followed by all selected studies. The majority of papers adhere to four core components, namely, comprehensive exercise, medical risk management, psychological management, and a multidisciplinary team. Conversely, lifestyle risk factor management and long-term strategy are the least addressed standards. Although nine out of 11 papers included a screening process to identify mental and psychological problems, only a limited number offered psychological support through face-to-face or online social interaction and conversation. A systematic review indicated a strong association between depression and both cardiac-related mortality and low participation in CR interventions [48]. Our review demonstrates that a majority of selected papers provide a comprehensive and individualized exercise program, including specified intensity, frequency, and duration based on the patient’s ability. However, chair-based exercise and simple walking are part of the exercise regimen in a minority of interventions. The review does not thoroughly explain the fidelity to prescribed physical activity. Fidelity to physical training may significantly decrease if participants lack alternative plans aligned with their preferences [49]. According to the ESC, individualized exercises based on the patient’s preferences and abilities contribute to long-term adherence and sustained health outcomes [26]. Our findings highlight a substantial lack of behavior change theories and lifestyle risk management in selected papers. Except for one paper offering nutritional guidance and advice [36], there is a notable absence of data on weight management, diet, and medication fidelity. Lifestyle interventions, as evidenced by a systematic review, result in a significant decrease in CVD risk factors compared to the control group [50].

The American College of Cardiology, the American Heart Association, and the American Association of Cardiovascular and Pulmonary Rehabilitation issued a scientific statement concerning patients with heart-related conditions to identify the core components, effectiveness, and limitations and needed research to improve the efficacy of delivering HBCR [20]. However, this paper paid no attention to important elements such as long-term strategy and recruiting a well-experienced team. A cross-sectional study on cardiac patients conducted in the US to evaluate the recommended standards and components for CR programs in accordance with the American Association of Cardiovascular and Pulmonary Rehabilitation (AACVPR) found that there is a lack of consistency in incorporating standards and core components in delivering CR interventions [51]. A previous systematic review and network meta-analysis (NMA) of randomized trials assessing the core components and standards of CR indicated that considering the core components of CR interventions, with an emphasis on exercise training as the most important component, leads to a substantial reduction in heart-related mortality and morbidity in patients with coronary heart disease (CHD) [52]. Furthermore, findings from a recent meta-analysis assessing patients with ischemic heart diseases illustrate a strong association between CR programs providing diverse core components and a significant reduction in cardiac-related fatality compared to those providing less [53]. Nevertheless, these papers did not provide an assessment of the proportion of CR intervention alliances with core components and standards.

In HF patients, since ICDs are self-managed and there is nothing that clinical change can do, assessing ICDs as a part of medical risk factor management is misleading. In this study, the best way to manage medical risk was medication management. Multiple drugs (beta-blockers, angiotensin-converting enzyme inhibitors, angiotensin receptor blockers, digoxin, loop diuretics, spironolactone, aspirin, anticoagulants, and statins) aimed at controlling BP, HR, and blood lipids are taken by HF patients, impacting numerous parts of the body. Therefore, by managing the medication used, it will be easier to assess other medical risks, including BP, HR, lipids, and glucose. Take patients using beta blockers as an example. These patients do not need monitoring for HR since beta blockers can control the dangerous increase in HR. The two guidelines (BACPR and ESC) used in this study are too generic (usually used in acute coronary syndrome, coronary revascularization, and HF patients); thus, this fact can be considered a potential critical comment.

## Limitations

This study is the first systematic search and critical review evaluating the extent to which HBCR interventions for HF patients align with clinical standards and core components for CR in accordance with both BACPR and ESC. We restricted our study to only papers in English, leaving out several papers (six studies). Hence, a comprehensive search with different languages should be considered to increase the generalizability of outcomes. We included HFrEF and excluded studies recruiting heart failure populations with preserved ejection fraction (HFpEF); therefore, considering patients with HFpEF based on our findings should be treated with caution.

### Suggestions for future research

This critical review has provided numerous opportunities for further research. First, conducting a review recruiting HFpEF and assessing the extent of their fidelity to core components. Second, assessing the proportion of CR alliance with core components of other guidelines such as AACVPR and the Australian Cardiovascular Health and Rehabilitation Association (ACRA) can be considered as other fields of study. Finally, there is a significant need for further research to specify the relationship between adherence to standards and components described by numerous cardiac guidelines and patients’ outcomes, including health behavior change, cardiac-related mortality, QOL, physical activity, and participation rates in CR programs. Eventually, future HBCR interventions are needed to allocate more emphasis to sharing their results with national audits, considering the long-term outcomes, treating patients suffering from mental problems, providing alternative exercise plans, and managing lifestyle risk factors.

## Conclusions

In summary, despite a low level of alliance with the long-term strategy component, HBCR interventions for HFrEF patients had mainly medium to high adherence to other core components and standards defined by both BACPR and ESC. Rapid identification and the initial assessment of patient needs are the most met standards; however, lifestyle risk factor management and assessing long-term outcomes were recognized as the least met standards. Strategies to ensure that future CR research study interventions incorporate risk factor management and assessment of long-term outcomes are needed.

### Supplementary Information

**Additional file 1.**

## Data Availability

The dataset supporting the conclusions of this article is included within the article.
