# Supplementary material for: Examining the effectiveness of home-based cardiac rehabilitation programs for heart failure patients with reduced ejection fraction: a critical review
Source: BMC Cardiovasc Disord. 2023 Dec 5;23:593. doi: 10.1186/s12872-023-03640-x (PMC10696730; doi:10.1186/s12872-023-03640-x)
Supplement: Supplementary file 1 — Additional file 1. [file 12872_2023_3640_MOESM1_ESM.pdf]

Appendix 1 Search Strategies for MEDLINE

Date range searched: 1946 to July 01, 2022

Date searched: 05 July 2022

Records retrieved: 1317

1 exp Heart Failure

2 ((heart* or cardiac or myocardial) adj (failure* or decompensation*)).ti,ab,kw.

3 (cardiac adj (edema* or oedema* or asthma)).ti,ab,kw.

4 ((cardio-renal or cardiorenal or cardio renal or reno-cardiac or renocardiac or reno cardiac) adj syndrome*).ti,ab,kw.

5 paroxysmal dyspnea*.ti,ab,kw.

6 or/1-5

7 Cardiac Rehabilitation

8 Heart Failure/rh

9 ((heart* or cardiac or myocardial) and (rehab* or exercis* or train* or therap*)).sh.

10 (heart* or cardiac or myocardial).sh. and rh.fs.

11 ((heart* or cardiac or myocardial) adj3 rehab*).ti,ab,kw.

12 or/7-11

13 6 and 12

14 randomized controlled trial.pt.

15 controlled clinical trial.pt.

16 randomized.ab.

17 placebo.ab.

18 drug therapy.fs.

19 randomly.ab.

20 trial.ab.

21 groups.ab.

22 or/14-21

23 exp animals/ not humans.sh.

24 22 not 23

25 13 and 24

26 "Delivery of Health Care"

27 (deliver* or hybrid* or mode*).ti,ab,kw.

28 home.hw.

29 (home* or home-based or domicil* or residential* or community or communities).ti,ab,kw.

30 tele*.sh.

31 Internet-Based Intervention

32 Internet

33 Cell Phone

34 Smartphone

35 Text Messaging

36 Mobile Applications

37 Wireless Technology

38 Computer Communication Networks

39 Social Media

40 Videoconferencing

41 Webcasts as Topic

42 (computer* or electronic* or digital* or technolog* or internet* or web or web based or web-based or web service* or web-service* or www or online or on-line or virtual* or wireless* or remote*).ti,ab,kw.

43 (tele* or i-health or iHealth or e-health or eHealth or electronic health or m-health or mHealth or mobile health).ti,ab,kw.

44 (mobile* or telecommunication* or tablet or telephone* or phone*1 or cell phone* or cell-phone* or cellphone or smartphone* or smart-phone* or iPhone or camera phone or camera-phone or handheld or hand-held or text messag* or text-messag* or texting or video call* or video-call* or videoconferenc* or video-conferenc* or video app or video-app or video imag* or video-imag* or still imag* or still-imag* or personal digital assistant or PDA or SMS or software or multi-media or multimedia).ti,ab,kw.

45 (android or app or apps or blog* or CD-ROM or chat room or cyber* or DVD or e-mail* or email* or e-Portal or ePortal or eTherap* or e-therap* or forum* or gaming or information technolog* or instant messag* or messaging or ipad or i-pad or iphone or i-phone or ipod or i-pod or podcast* or social network* or social medi*).ti,ab,kw.

46 or/26-45

47 25 and 46

48 remove duplicates from 47

Key:

/ or .sh. = indexing term (Medical Subject Heading: MeSH)

exp = exploded indexing term (MeSH)

/rh = indexing term with rehabilitation subheading

* = unlimited truncation

ti,ab,kw = terms in either title, abstract, keyword fields

adj3 = terms within three words of each other (any order)

? = optional wild card character for zero or one letters

pt = publication type

fs = floating subheading

Appendix 2 Search Strategies for Embase

Date range searched: 1974 to 2022 July 01

Date searched: 05 July 2022

Records retrieved: 2462

1 exp heart failure

2 ((heart* or cardiac or myocardial) adj (failure* or decompensation*)).ti,ab,kw.

3 (cardiac adj (edema* or oedema* or asthma)).ti,ab,kw.

4 ((cardio-renal or cardiorenal or cardio renal or reno-cardiac or renocardiac or reno cardiac) adj syndrome*).ti,ab,kw.

5 paroxysmal dyspnea*.ti,ab,kw.

6 or/1-5

7 heart rehabilitation

8 heart failure/rh

9 ((heart* or cardiac or myocardial) and (rehab* or exercis* or train* or therap*)).sh.

10 (heart* or cardiac or myocardial).sh. and rh.fs.

11 ((heart* or cardiac or myocardial) adj3 rehab*).ti,ab,kw.

12 or/7-11

13 6 and 12

14 randomized controlled trial

15 controlled clinical trial

16 Random$.ti,ab,ot.

17 randomization

18 intermethod comparison

19 placebo.ti,ab,ot.

20 (compare or compared or comparison).ti,ot.

21 ((evaluated or evaluate or evaluating or assessed or assess) and (compare or compared or comparing or comparison)).ab.

22 (open adj label).ti,ab,ot.

23 ((double or single or doubly or singly) adj (blind or blinded or blindly)).ti,ab,ot. (257935)

24 double blind procedure

25 parallel group$1.ti,ab,ot.

26 (crossover or cross over).ti,ab,ot.

27 ((assign$ or match or matched or allocation) adj5 (alternate or group or groups or intervention or interventions or patient or patients or subject or subjects or participant or participants)).ti,ab,ot.

28 (assigned or allocated).ti,ab,ot.

29 (controlled adj7 (study or design or trial)).ti,ab,ot.

30 (volunteer or volunteers).ti,ab,ot.

31 human experiment

32 trial.ti,ot.

33 or/14-32

34 13 and 33

35 (rat or rats or mouse or mice or swine or porcine or murine or sheep or lambs or pigs or piglets or rabbit or rabbits or cat or cats or dog or dogs or cattle or bovine or monkey or monkeys or trout or marmoset$).ti,ot. and animal experiment

36 Animal experiment/ not (human experiment/ or human/)

37 35 or 36

38 34 not 37

39 health care delivery

40 (deliver* or hybrid* or mode*).ti,ab,kw.

41 home.hw.

42 (home* or home-based or domicil* or residential* or community or communities).ti,ab,kw.

43 tele*.sh.

44 web-based intervention

45 Internet

46 mobile phone

47 smartphone

48 text messaging

49 mobile application

50 wireless communication

51 computer network

52 social media

53 videoconferencing

54 webcast

55 (computer* or electronic* or digital* or technolog* or internet* or web or web based or web-based or web service* or web-service* or www or online or on-line or virtual* or wireless* or remote*).ti,ab,kw.

56 (tele* or i-health or iHealth or e-health or eHealth or electronic health or m-health or mHealth or mobile health).ti,ab,kw.

57 (mobile* or telecommunication* or tablet or telephone* or phone*1 or cell phone* or cell-phone* or cellphone or smartphone* or smart-phone* or iPhone or camera phone or camera-phone or handheld or hand-held or text messag* or text-messag* or texting or video

call* or video-call* or videoconferenc* or video-conferenc* or video app or video-app or video imag* or video-imag* or still imag* or still-imag* or personal digital assistant or PDA or SMS or software or multi-media or multimedia).ti,ab,kw.

58 (android or app or apps or blog* or CD-ROM or chat room or cyber* or DVD or e-mail* or email* or e-Portal or ePortal or eTherap* or e-therap* or forum* or gaming or information technolog* or instant messag* or messaging or ipad or i-pad or iphone or i-phone or ipod or i-pod or podcast* or social network* or social medi*).ti,ab,kw.

59 or/39-58

60 38 and 59

61 letter/ or case report/ or case study

62 (letter or comment*).ti.

63 (comment or conference or letter or editorial or note).pt.

64 or/61-63

65 60 not 64

66 remove duplicates from 65

Key:

/ or .sh. = indexing term (Emtree subject heading)

exp = exploded indexing term (Emtree)

* = unlimited truncation

/rh = indexing term with rehabilitation subheading

ti,ab,kw = terms in either title, abstract or keyword fields

adj3 = terms within three words of each other (any order)

? = optional wild card character for zero or one letters

ot = original title

pt = publication type

Appendix 3 Search Strategies for Cochrane Central Register of Controlled Trials (CENTRAL)

Date range: Issue 7 of 12, July 2022

Date searched: 05 July 2022

Records retrieved: 488

#1 [mh "Heart Failure"]c

#2 ((heart* or cardiac or myocardial) NEXT (failure* or decompensation*)):ti,ab,kw

#3 (cardiac NEXT (edema* or oedema* or asthma)):ti,ab,kw

#4 ((cardio NEXT renal or cardiorenal or reno NEXT cardiac or renocardiac) NEXT syndrome*):ti,ab,kw

#5 paroxysmal NEXT dyspnea*:ti,ab,kw

#6 #1 OR #2 OR #3 OR #4 OR #5

#7 [mh ^"Cardiac Rehabilitation"]

#8 [mh ^"Heart Failure"/rh]

#9 ((heart* or cardiac or myocardial) NEAR/3 rehab*):ti,ab,kw

#10 #7 OR #8 OR #9

#11 #6 AND #10

#12 [mh ^"health care delivery"]

#13 [mh ^"Home Environment"]

#14 [mh ^"Home Care Services"]

#15 [mh ^"Home Health Nursing"]

#16 [mh Telemedicine]

#17 [mh ^"web-based intervention"]

#18 [mh ^"internet"]

#19 [mh ^"mobile phone"]

#20 [mh ^smartphone]

#21 [mh ^"text messaging"]

#22 [mh ^"mobile application"]

#23 [mh ^"wireless communication"]

#24 [mh ^"computer network"]

#25 [mh ^"social media"]

#26 [mh ^videoconferencing]

#27 [mh ^webcast]

#28 (deliver* or hybrid* or mode*):ti,ab,kw

#29 (home* or domicil* or residential* or community or communities):ti,ab,kw

#30 (computer* or electronic* or digital* or technolog* or internet* or web or web NEXT based or web NEXT service* or www or online or on NEXT line or virtual* or wireless* or remote*):ti,ab,kw

#31 (tele* or i NEXT health or iHealth or e NEXT health or eHealth or electronic NEXT health or m NEXT health or mHealth or mobile NEXT health):ti,ab,kw

#32 (mobile* or telecommunication* or tablet or telephone* or phone* or cell NEXT phone* or cellphone or smartphone* or smart NEXT phone* or iPhone or camera NEXT phone or handheld or hand NEXT held or text NEXT messag* or texting or video NEXT call* or videoconferenc* or video NEXT conferenc* or video NEXT app or video NEXT imag* or still NEXT imag* or "personal digital assistant" or PDA or SMS or software or multi NEXT media or multimedia):ti,ab,kw

#33 (android or app or apps or blog* or CD NEXT ROM or chat NEXT room or cyber* or DVD or e NEXT mail* or email* or e NEXT Portal or ePortal or eTherap* or e NEXT therap* or forum* or gaming or information technolog* or instant NEXT messag* or messaging or ipad or i NEXT pad or iphone or i NEXT phone or ipod or i NEXT pod or podcast* or social NEXT network* or social NEXT medi*):ti,ab,kw

#34 (Hwang et al.-#33)

#35 #11 AND #34 in Trials

Key:

mh = exploded indexing term (MeSH)

mh ^ = unexploded indexing term (MeSH)

/rh = indexing term with rehabilitation subheading

* = truncation

ti,ab,kw = terms in either title or abstract or keyword fields

near/3 = terms within three words of each other (any order)

next = terms are next to each other

Appendix 4 Search Strategies for Cochrane Databases of Systematic Reviews (CDSR)

Date range: Issue 7 of 12, July 2022

Date searched: 05 July 2022

Records retrieved: 5

#1 [mh "Heart Failure"]

#2 ((heart* or cardiac or myocardial) NEXT (failure* or decompensation*)):ti,ab,kw

#3 (cardiac NEXT (edema* or oedema* or asthma)):ti,ab,kw

#4 ((cardio NEXT renal or cardiorenal or reno NEXT cardiac or renocardiac) NEXT syndrome*):ti,ab,kw

#5 paroxysmal NEXT dyspnea*:ti,ab,kw

#6 #1 OR #2 OR #3 OR #4 OR #5

#7 [mh ^"Cardiac Rehabilitation"]

#8 [mh ^"Heart Failure"/rh]

#9 ((heart* or cardiac or myocardial) NEAR/3 rehab*):ti,ab,kw

#10 #7 OR #8 OR #9

#11 #6 AND #10

#12 [mh ^"health care delivery"]

#13 [mh ^"Home Environment"]

#14 [mh ^"Home Care Services"]

#15 [mh ^"Home Health Nursing"]

#16 [mh Telemedicine]

#17 [mh ^"web-based intervention"]

#18 [mh ^"internet"]

#19 [mh ^"mobile phone"]

#20 [mh ^smartphone]

#21 [mh ^"text messaging"]

#22 [mh ^"mobile application"]

#23 [mh ^"wireless communication"]

#24 [mh ^"computer network"]

#25 [mh ^"social media"]

#26 [mh ^videoconferencing]

#27 [mh ^webcast]

#28 (deliver* or hybrid* or mode*):ti,ab,kw

#29 (home* or domicil* or residential* or community or communities):ti,ab,kw

#30 (computer* or electronic* or digital* or technolog* or internet* or web or web NEXT based or web NEXT service* or www or online or on NEXT line or virtual* or wireless* or remote*):ti,ab,kw

#31 (tele* or i NEXT health or iHealth or e NEXT health or eHealth or electronic NEXT health or m NEXT health or mHealth or mobile NEXT health):ti,ab,kw

#32 (mobile* or telecommunication* or tablet or telephone* or phone* or cell NEXT phone* or cellphone or smartphone* or smart NEXT phone* or iPhone or camera NEXT phone or handheld or hand NEXT held or text NEXT messag* or texting or video NEXT

call* or videoconferenc* or video NEXT conferenc* or video NEXT app or video NEXT imag* or still NEXT imag* or "personal digital assistant" or PDA or SMS or software or multi NEXT media or multimedia):ti,ab,kw

#33 (android or app or apps or blog* or CD NEXT ROM or chat NEXT room or cyber* or DVD or e NEXT mail* or email* or e NEXT Portal or ePortal or eTherap* or e NEXT therap* or forum* or gaming or information technolog* or instant NEXT messag* or messaging or ipad or i NEXT pad or iphone or i NEXT phone or ipod or i NEXT pod or podcast* or social NEXT network* or social NEXT medi*):ti,ab,kw

#34 (Hwang et al.-#33)

#35 #11 AND #34 in Cochrane Reviews

Key:

mh = exploded indexing term (MeSH)

mh ^ = unexploded indexing term (MeSH)

/rh = indexing term with rehabilitation subheading

* = truncation

ti,ab,kw = terms in either title or abstract or keyword fields

near/3 = terms within three words of each other (any order)

next = terms are next to each oth

Appendix 5 Version 2 of the Cochrane Risk-of-bias assessment tool for randomized trials

Bias domain	Signaling question	Response options*
**Bias arising from the randomization process**	1.1 Was the allocation sequence random?	Y/PY/PN/N/NI
	1.2 Was the allocation sequence concealed until participants were enrolled and assigned to interventions?	Y/PY/PN/N/NI
	1.3 Did baseline differences between intervention groups suggest a problem with the randomization process?	Y/PY/PN/N/NI
	**Risk of bias judgement** (Low/High/Some concerns)	
	Optional: What is the predicted direction of bias arising from the randomization process?	
**Bias due to deviations from intended interventions**	2.1. Were participants aware of their assigned intervention during the trial?	Y/PY/PN/N/NI
	2.2. Were carers and people delivering the interventions aware of participants' assigned intervention during the trial?	Y/PY/PN/N/NI
	2.3. If Y/PY/NI to 2.1 or 2.2: Were there deviations from the intended intervention that arose because of the experimental context?	NA/Y/PY/PN/N/NI
	2.4 If Y/PY/NI to 2.3: Were these deviations likely to have affected the outcome?	NA/Y/PY/PN/N/NI
	2.5. If Y/PY to 2.4: Were these deviations from intended intervention balanced between groups?	NA/Y/PY/PN/N/NI
	2.6 Was an appropriate analysis used to estimate the effect of assignment to intervention?	Y/PY/PN/N/NI
	2.7 If N/PN/NI to 2.6: Was there potential for a substantial impact (on the result) of the failure to analyze participants in the group to which they were randomized?	NA/Y/PY/PN/N/NI
	**Risk of bias judgement** (Low/High/Some concerns)	
	Optional: What is the predicted direction of bias due to deviations from intended interventions?	
**Bias due to missing outcome data**	3.1 Were data for this outcome available for all, or nearly all, participants randomized?	Y/PY/PN/N/NI
	3.2 If N/PN/NI to 3.1: Is there evidence that the result was not biased by missing outcome data?	NA/Y/PY/PN/N
	3.3 If N/PN to 3.2: Could missingness in the outcome depend on its true value?	NA/Y/PY/PN/N/NI
	3.4 If Y/PY/NI to 3.3: Is it likely that missingness in the outcome depended on its true value?	NA/Y/PY/PN/N/NI

	**Risk of bias judgement** (Low/High/Some concerns)	
	Optional: What is the predicted direction of bias due to missing outcome data?	
**Bias in measurement of the outcome**	4.1 Was the method of measuring the outcome inappropriate?	Y/PY/PN/N/NI
	4.2 Could measurement or ascertainment of the outcome have differed between intervention groups?	Y/PY/PN/N/NI
	4.3 If N/PN/NI to 4.1 and 4.2: Were outcome assessors aware of the intervention received by study participants?	Y/PY/PN/N/NI
	4.4 If Y/PY/NI to 4.3: Could assessment of the outcome have been influenced by knowledge of intervention received?	NA/Y/PY/PN/N/NI
	4.5 If Y/PY/NI to 4.4: Is it likely that assessment of the outcome was influenced by knowledge of intervention received?	NA/Y/PY/PN/N/NI
	**Risk of bias judgement** (Low/High/Some concerns)	
	Optional: What is the predicted direction of bias in measurement of the outcome?	
**Bias in selection of the reported result**	5.1 Were the data that produced this result analyzed in accordance with a pre-specified analysis plan that was finalised before unblinded outcome data were available for analysis?<br>Is the numerical result being assessed likely to have been selected, on the basis of the results, from...	Y/PY/PN/N/NI
	5.2. ... multiple eligible outcome measurements (e.g., scales, definitions, time points) within the outcome domain?	Y/PY/PN/N/NI
	5.3 ... multiple eligible analyses of the data?	Y/PY/PN/N/NI
	**Risk of bias judgement** (Low/High/Some concerns)	
	Optional: What is the predicted direction bias due to selection of the reported results?	
**Overall bias**	**Risk of bias judgement** (Low/High/Some concerns)	
	Optional: What is the overall predicted direction of bias for this outcome?	

* Y: yes; PY: probably yes; PN: probably no; N: no; NA: not applicable; NI: no information. Responses in green and underlined correspond to lower risk of bias. Responses in red correspond to higher risk of bias
